# Unilateral Acute Anterior Ischemic Optic Neuropathy in a Patient with an Already Established Diagnosis of Bilateral Optic Disc Drusen

**DOI:** 10.1155/2015/730606

**Published:** 2015-10-15

**Authors:** Ziya Ayhan, Aylin Yaman, Meltem Söylev Bajin, A. Osman Saatci

**Affiliations:** Department of Ophthalmology, Dokuz Eylül University, 35340 İzmir, Turkey

## Abstract

Optic disc drusen (ODD) are calcific deposits that form in the optic nerve head secondary to abnormalities in axonal metabolism and degeneration. Anterior ischemic optic neuropathy, central retinal artery, and vein occlusion are among the rare vascular complications of disc drusen. We reported the clinical course of a 51-year-old patient with a unilateral acute nonarteritic anterior ischemic optic neuropathy (NAION) who received the diagnosis of bilateral optic disc drusen five years earlier and thereby reiterated the association of ODD and acute NAION.

## 1. Introduction

Optic disc drusen (ODD) are calcific deposits that form in the optic nerve head secondary to abnormalities in axonal metabolism and degeneration and are found in 0.4 to 3.7% of the population [1, 2]. They may be seen as reflective bodies emanating through the disc or may be buried imitating papilledema [1, 2]. Most patients with ODD are asymptomatic and diagnosed coincidentally in a routine eye examination. However, visual field defects may occur and be detected in up to 90% of the cases [1–3]. Severe visual impairment can very occasionally develop due to vascular complications such as acute nonarteritic ischemic optic neuropathy (NAION), central retinal artery, and vein occlusion in patients with ODD [4–9]. In this report, we report a case of acute NAION in a patient with an already established diagnosis of ODD.

## 2. Case Report

**Figure 1:**
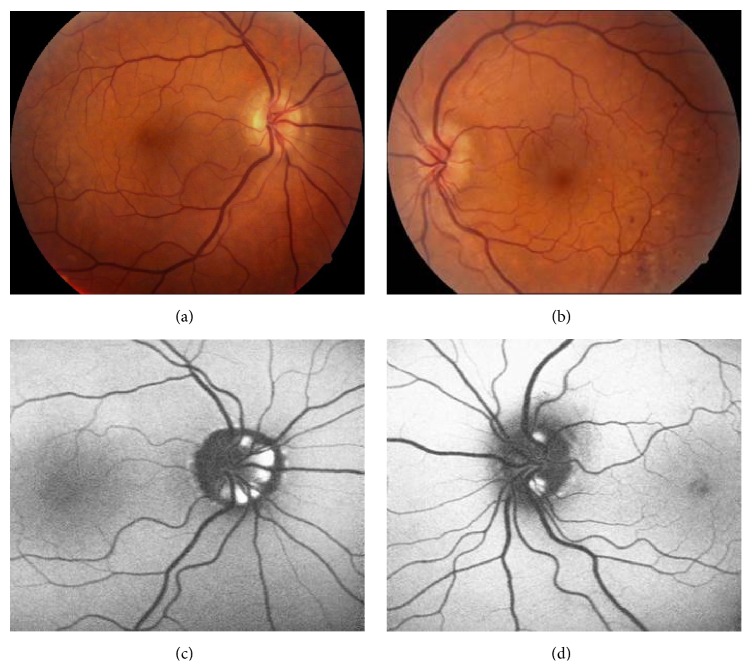
Clinical appearance of both posterior poles in 2009. Color fundus pictures depicting the optic disc drusen (a) right eye; (b) left eye and fundus autofluorescent pictures depicting the hyperautofluorescence due to disc drusen, (c) right eye; (d) left eye.

A 46-year-old man was examined by us for minor ocular problems in January 2009. He had received a diagnosis of type 2 diabetes mellitus five years priorly and has been known to have congenital color blindness. At that time, his best-corrected visual acuity (BCVA) with Snellen chart was 8/10 in OD and 7/10 in OS. Slit-lamp examination was normal OU. Fundus examination and autofluorescent imaging showed bilateral mild nonproliferative diabetic retinopathy and optic disc drusen (Figures 1(a), 1(b), 1(c), and 1(d)).

The patient was further referred to the endocrinology department for blood sugar regulation. However, the patient was lost to the follow-up.

**Figure 2:**
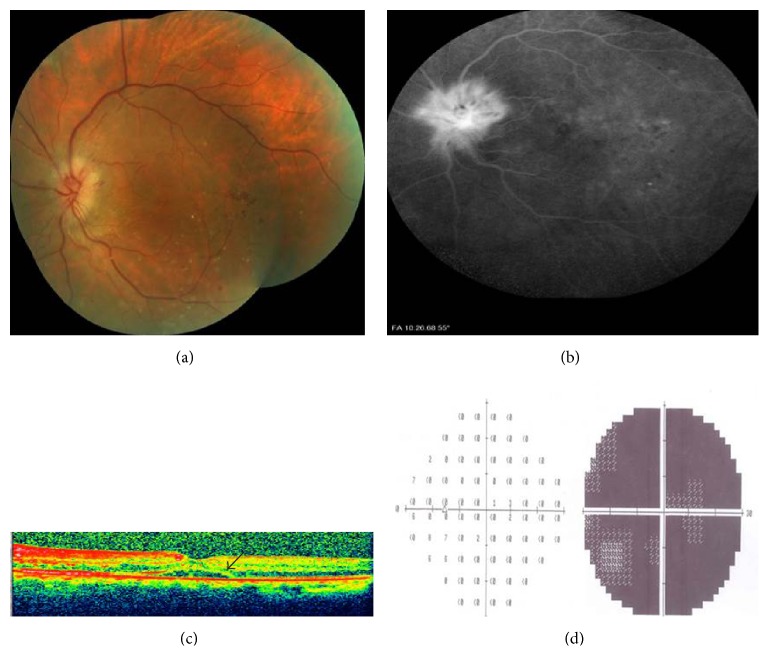
Left eye, 2 days after the painless visual loss; (a) color fundus picture showing the segmentally swollen disc; (b) late venous phase of angiography depicting the disc leakage; (c) optic coherence tomography revealing the subretinal fluid (arrow); (d) visual field examination demonstrating the severe diffuse depression.

He returned to the eye clinic with a sudden painless visual loss in his left eye of two days' duration in October 2014. On our examination, left afferent pupillary defect was present. Color vision was 7/21 in OD and 1/21 in OS with Ishihara pseudoisochromatic plates. Visual acuity was 8/10 in OD and 1/10 in OS. Slit-lamp examination was unremarkable OU. Intraocular pressure was within normal limit bilaterally. While right optic disc had only drusen and no physiologic cup, left optic disc also was hyperemic and segmentally swollen and a peripapillary splinter haemorrhage was present (Figure 2(a)). The laboratory tests, including complete blood count, erythrocyte sedimentation rate, and CRP, were normal. There was prominent fluorescein leakage at the left optic disc (Figure 2(b)). OCT disclosed mild subretinal fluid in OS (Figure 2(c)) and no abnormality in OD. Visual field testing disclosed inferior altitudinal scotoma in OD most possibly in association with disc drusen and severe diffuse depression in OS (Figure 2(d)).

**Figure 3:**
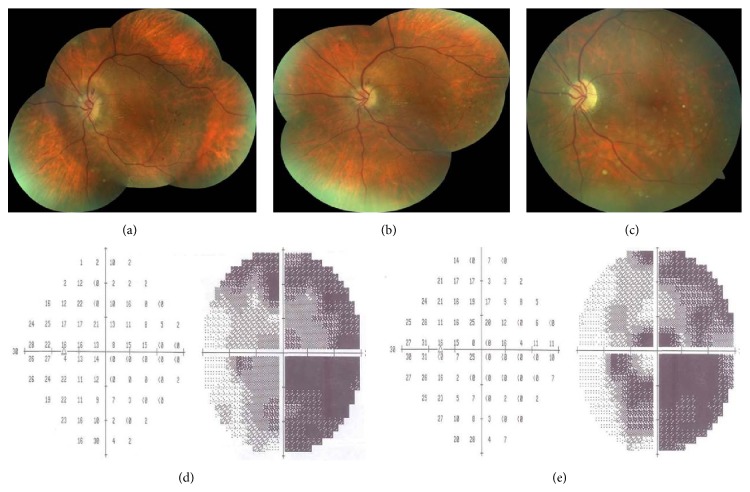
Left eye, color fundus pictures taken at the first week (a), first month (b), and third month after the injection (c) showing the resolution of the disc edema with subsequent occurrence of pallor. Visual field test demonstrating the gradual improvement at the first (d) and third month (e).

Our diagnosis was left acute NAION with ODD. Natural course, treatment options of the acute NAION, and our preference for intravitreal injection of either ranibizumab or triamcinolone acetonide based on our previous retrospective studies [10, 11] were discussed with the patient and 0.5 mg ranibizumab was injected intravitreally into the left eye next day. Best-corrected visual acuity was 2/10 a week later, 6/10 at the first month, and 9/10 at the third month in OS. He could read 1/21 of plates a week later and 2/21 of plates at the first and third month with Ishihara test. Color fundus pictures of the left eye taken at the first week and first and third month after the intravitreal injection are shown in Figures 3(a), 3(b), and 3(c). There was a partial improvement in the visual field at the end of third month in OS. Visual fields obtained at the first and third posttreatment month are shown in Figures 3(d) and 3(e). No injection related complication was noted during the follow-up of three months.

## 3. Discussion

It was suggested that a small disk area along with a horizontal shortening of the scleral canal could lead to the crowding of optic nerve fibers, predisposing to a circulatory compromise of the optic nerve head in acute NAION [12–14]. Furthermore, Mullie and Sanders [15] measured the size of the scleral canal from projected optic disc photographs in two samples of emmetropic patients: one of patients with unilateral pseudopapilledema and drusen and the other of the general normal population. Measurements on the non-drusen-containing optic disk of patients with unilateral drusen were taken to reflect the canal size of the affected eye. The average diameters of the non-drusen-containing optic disk of patients with unilateral drusen were significantly smaller than those of the optic disk of normal patients. They concluded that the association of a small scleral canal with vascular anomalies noted in optic disk of patients with drusen indicated a mesodermal dysgenesis of the optic nerve head.

Mostly anecdotal case reports with acute NAION and concomitant optic disc drusen were present in the literature [16–25]. Gittinger et al. [16] reported five eyes of four patients with acute NAION associated with ODD. They speculated that infarction of the distal portion of the optic nerve in patients with drusen might result from the mechanical distortion of blood vessels located in the laminar and prelaminar regions. Purvin et al. [26] reviewed the medical reports of 20 patients with ODD and acute NAION and compared them with the data from previously reported series of patients with acute NAION. Study patients were strikingly similar to those with acute NAION unassociated with drusen in regard to prevalence of vascular risk factors, pattern of visual field loss, and occurrence of subsequent similar event in the fellow eye. In contrast, patients with ODD-NAION were younger than those with pure acute NAION, were more likely to report preceding episodes of transient visual obscuration, and enjoyed a more favorable visual outcome.

Present case was 51 years old and had type 2 diabetes that could be considered as a predisposing factor for the development of acute NAION. Our case had also a very good visual outcome following the treatment as concluded by the Purvin and colleagues [26]. This result might be due to our treatment or attributed to the natural history. To our best knowledge, the present case is the only case in the literature that had previous documentation of optic disc drusen and five years later developed unilateral acute NAION. In light of our case and literature survey, we once again underlined the importance of recognition of the presence of optic disc drusen in cases with acute NAION.
